# Nutritional Status of Vitamin E and Its Association with Metabolic Health in Adults

**DOI:** 10.3390/nu17030408

**Published:** 2025-01-23

**Authors:** Kacper Szewczyk, Joanna Bryś, Rita Brzezińska, Magdalena Górnicka

**Affiliations:** 1 Department of Human Nutrition, Institute of Human Nutrition Sciences, Warsaw University of Life Sciences, Nowoursynowska St. 166, 02-787 Warsaw, Poland; kacper_szewczyk@sggw.edu.pl; 2 Department of Chemistry, Institute of Food Sciences, Warsaw University of Life Sciences, Nowoursynowska St. 159c, 02-787 Warsaw, Poland; joanna_brys@sggw.edu.pl (J.B.); rita_brzezinska@sggw.edu.pl (R.B.)

**Keywords:** plasma α-tocopherols, plasma γ-tocopherols, plasma α-tocotrienols, plasma γ-tocotrienols, body fat, plasma fatty acids, lipid status, inflammation, adults

## Abstract

Background: Vitamin E is one of the key dietary antioxidants. However, current evidence remains insufficient to establish a definitive relationship between circulating vitamin E levels, body fat content, and their influence on metabolic health. This study aimed to assess and compare the vitamin E nutritional status in adults with normal and excess body fat and its determinants. Methods: Concentrations of vitamin E isoforms (α- and γ-tocopherols, α- and γ-tocotrienols) were assessed in 127 individuals. Body fat content and other anthropometric indices, as well as biochemical markers such as lipid profile, plasma fatty acid concentration and C-reactive protein, were identified as markers of metabolic health. Participants were divided into two groups: with normal and excess body fat (defined as more than 30% in women and more than 25% in men). Results: The determinants of higher α-tocopherol concentrations were lower body fat content and higher levels of circulating lipids as HDL and LDL (R^2^ = 0.221, *p* < 0.001 in a model of multivariate linear regression). The level of circulating vitamin E isoforms correlated with the concentration of CRP (r = −0.464 for α-T, r = −0.453 for αT3, r = −0.270 for γ-T, r = −0.355 for γ-T3). Similarly, elevated concentrations of vitamin E isoforms are linked to lower adipose tissue content, which may contribute to lower inflammation and improved metabolic health (r = −0.359 for α-T, r = −0.333 for αT3, r = −0.276 for γ-T3, no significant correlation for γ-T). Conclusions: These results reveal that the vitamin E status of adults with excess body fat may be inadequate and linked to poorer metabolic health. We found that the determinants of lower plasma vitamin E were higher BF and lower TC and its fraction, with the strongest correlations being found for HDL.

## 1. Introduction

Vitamin E is a group of eight structurally similar compounds comprising four tocopherols (α-; β-; γ-; δ-forms) with single bonds between carbon atoms and four tocotrienols (α-; β-; γ-; δ-forms), each featuring a single double bond in the side chain [1]. This family of compounds, classified as vitamin E, is synthesized by plants and is primarily found in dietary sources such as vegetable oils, seeds, kernels, and nuts [2]. Current findings suggest that various forms of vitamin E may protect cell membranes from oxidative damage, contribute to the inhibition of inflammatory pathways leading to atherosclerosis and non-alcoholic fatty liver disease, and reduce inflammatory biomarkers in individuals with insulin resistance [3,4]. Vitamin E plays a multifaceted role in adipose tissue, influencing its metabolic and structural characteristics. Evidence suggests that vitamin E may modulate adipose tissue function by reducing oxidative stress, inflammation, and fibrosis, thereby improving metabolic profiles in the context of obesity [5]. Circulating levels of vitamin E, particularly α- and γ-tocopherol, have been positively associated with increased visceral and subcutaneous adipose tissue volumes, as well as a higher likelihood of metabolic syndrome. This relationship indicates a complex interplay in which vitamin E levels correlate with specific features of excess body fat [6].

Results of our previous studies have shown that only 40–57% of the studied group met the level of adequate intake (AI) for vitamin E [7]. The predominant dietary forms were α- and γ-tocopherols and tocotrienols [4]. Among these, α-tocopherol is the most biologically active and extensively studied due to the presence of a specific transport protein, α-tocopherol transfer protein (α-TTP). However, research suggests that other forms of vitamin E may be preferentially absorbed or utilized when circulating α-tocopherol levels are low [8].

In recent years, other forms of vitamin E have gained attention for their superior antioxidant and anti-inflammatory properties compared to α-tocopherol, leading to a shift in research focus [4,7,9]. Tocotrienols are also believed to promote angiogenesis and regulate enzyme activity and transcriptional pathways, which may contribute to cancer prevention [10]. Vitamin E has shown also promising results in reducing inflammation measured by the C-reactive protein (CRP) level and/or tumor necrosis factor-α (TNF-α) concentration, suggesting its potential as a therapeutic agent in the treatment of inflammation-related diseases [4].

Vitamin E is absorbed in the small intestine, often enhanced by the presence of dietary fats, and is incorporated into chylomicrons for transport through the lymphatic system [11]. In the liver, α-tocopherol is preferentially incorporated into very low-density lipoproteins (VLDL) and secreted into the bloodstream [12]. Vitamin E is distributed in the plasma primarily associated with lipoproteins, including low-density lipoproteins (LDL) and high-density lipoproteins (HDL) [13]. The absorption of vitamin E is increased when consumed with dietary fat. Low-fat diets may result in reduced vitamin E levels within the body [14].

Thus, vitamin E metabolism is associated with dietary lipids (found in vegetable oils), body lipids (transported by HDL and magazine in fat tissue) and cell lipids (cell membranes) [15]. The bioavailability and utilization of vitamin E are influenced by many age-related factors, including dietary intake, absorption, transport, and metabolism [16].

Plasma α-tocopherol is commonly used as a marker of vitamin E status, despite the lack of a definitive correlation between its plasma levels and tissue concentrations [17]. Since α-tocopherol is primarily associated with lipoproteins in the lipid fraction, minor fluctuations in circulating lipids can significantly affect its plasma concentration. Therefore, lipid-adjusted α-tocopherol—normalized to cholesterol or triglycerides—is considered a more reliable indicator [18]. Although plasma concentrations of vitamin E in older adults are comparable to those in younger adults, older adults have increased oxidative stress and subsequent cellular damage. This is thought to be one of the mechanisms underlying the age-related dysregulation of immune and inflammatory responses, resulting in increased incidence, morbidity, and mortality from infections and chronic noncommunicable diseases [16].

The relationship between fatty acid profiles and vitamin E availability primarily arises from their common dietary sources, such as oils and nuts, which are rich in both nutrients [19,20]. These sources provide essential nutrients, including polyunsaturated fatty acids (PUFAs) and vitamin E, with the latter’s concentration increasing in parallel with the levels of unsaturated fatty acids [19]. The coexistence of these nutrients in dietary sources highlights a potential relationship between their intake and associated health benefits [20]. Fatty acids in plasma serve as biomarkers for short-term dietary fat intake, exhibiting rapid responses to changes in dietary composition [21]. High-fat diets significantly influence lipid management parameters by altering the concentrations of total cholesterol, LDL cholesterol, and triglycerides, thereby modifying the overall lipid profile in the bloodstream [22].

Moreover, emerging evidence suggests that circulating fatty acids, a cause of oxidative stress, are related to metabolic disorders and could play a role in obesity [23,24]. The inflammatory potential of fatty acids in obesity varies significantly depending on their type, with distinct effects on inflammation and metabolic health. Saturated fatty acids (SFA) are generally pro-inflammatory, whereas omega-3 polyunsaturated fatty acids (PUFA) and monounsaturated fatty acids (MUFA) exhibit anti-inflammatory properties [25,26]. Dietary fats are critical determinants of plasma lipid concentrations and play a central role in lipid metabolism [27]. Among these, non-esterified fatty acids (NEFAs), often referred to as fatty acids, are of particular interest due to their involvement in obesity-related metabolic dysfunction [28].

The metabolism of fatty acids is closely linked to the activity of key enzymes, including delta-5 desaturase (D5D), delta-6 desaturase (D6D), and stearoyl-CoA desaturases (SCD-16 and SCD-18), which play crucial roles in metabolic health. D5D activity is generally associated with favorable metabolic effects, such as a reduced risk of metabolic syndrome (MetS), lower triglyceride levels, decreased diastolic blood pressure, and reduced waist circumference [29]. The activities of D6D, SCD-16 and SCD-18 are associated with adverse metabolic outcomes, such as elevated triglyceride levels and reduced HDL cholesterol, contributing to the development of MetS [29,30,31].

The relationship between plasma vitamin E isoforms and metabolic health is not yet fully understood. In our study, we considered ’good’ metabolic health as the absence of the metabolic syndrome components [32] and lower risk of cardiometabolic diseases, evaluated through higher D5D and lower D6D, SCD-16, SCD-18 activity, as well as decreased levels of inflammation. Therefore, the aim of this study was to assess and compare the nutritional status of vitamin E in adults with normal and excess body fat content and its determinants. Furthermore, it examined the hypothesis that lower concentrations of α-tocopherol, γ-tocopherol, α-tocotrienol, and γ-tocotrienol are linked with poorer metabolic health (including excess body fat and central adiposity, abnormal profile of lipid compounds, and elevated inflammation).

## 2. Materials and Methods

### 2.1. Ethical Approval

The research adhered to the principles outlined in the Declaration of Helsinki, and the Ethics Committee of the Institute of Human Nutrition Sciences of the Warsaw University of Life Sciences (Resolution No. 05/2019), approved all procedures involving human subjects. Before participating in the study, all subjects provided their written informed consent, before the survey.

### 2.2. Individuals Recruitment

**Figure 1:**
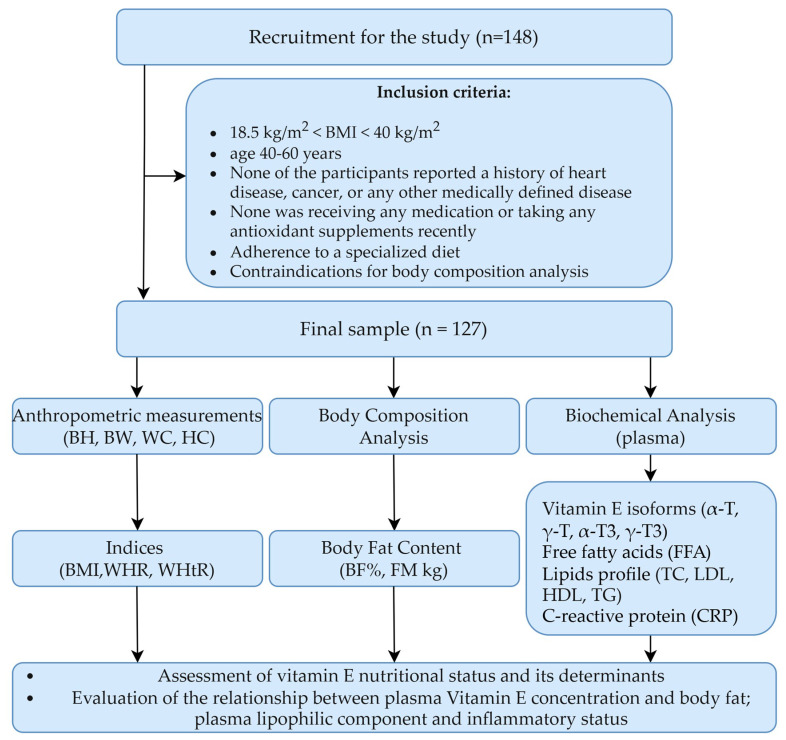
Study design. BH, body height; BW, body weight; WC, waist circumference; HC, hip circumference; BF, body fat content; BMI, body mass index; WHR, waist–hip ratio; WHtR, waist to height ratio; FM, fat mass; T, tocopherol; T3, tocotrienol; FA, fatty acids; TC, total cholesterol; LDL, low-density lipoprotein; HDL, high-density lipoprotein; TG, triacylglycerols; CRP, C-reactive protein.

The recruitment for this observational, cross-sectional study took place between October 2021 and October 2022. Participants were recruited from social media users through advertisements in social media groups. Patients came from both the Warsaw agglomeration and other towns from the entire territory of Poland. A total of 148 people expressed their willingness to participate in the study. The study involved one meeting where anthropometric data, blood samples, and sociodemographic data were collected. The study group selection process and the inclusion criteria are outlined in Figure 1. After applying the criteria, the data from 127 subjects were included in the study.

### 2.3. Anthropometric Measurements

Anthropometric measurements, including body height (BH), body weight (BW), waist circumference (WC), and hip circumference (HC), were conducted following the standardized protocols outlined in the International Standards for Anthropometric Assessment (ISAK) guidelines [33]. The measurements employed professional-grade equipment and a calibrated measuring tape. Body mass was assessed using a digital electronic scale with a precision of 0.1 kg (SECA 799, Hamburg, Germany). BH was determined with a stadiometer, ensuring the head was positioned in the Frankfurt horizontal plane, and recorded with an accuracy of 0.1 cm (SECA 220, Hamburg, Germany). WC was measured using a stretch-resistant tape that applies a constant tension of 100 g (SECA 201, Hamburg, Germany), positioned midway between the iliac crest and the lower rib at the anterior axillary line during a relaxed exhalation. HC was recorded at the widest part of the buttocks, with the measuring tape held parallel to the floor.

Based on the anthropometric measurements, several key anthropometric indices commonly used in screening studies were calculated: the Body Mass Index (BMI) calculated as body mass in kilograms divided by the square of body height in meters (kg/m^2^). The range from 18.5 to 24.99 was considered a normal body weight, whereas a BMI above 25 was classified as overweight [34]. Additionally, the waist-to-hip ratio (WHR) and the waist-to-height ratio (WHtR) were computed, serving as indices of central obesity and metabolic risk assessment. WHR above 0.8 for women and men was interpreted as an accumulation of adipose tissue in the android area, while the opposite results as the accumulation of adipose tissue in the gynoid area [34]. For WHtR, a cut-off point of 0.5 was adopted, above which results indicate excess adipose tissue accumulation in the abdominal area [34].

### 2.4. Body Composition Analysis

Body composition, including Body Fat Content (BF) and Fat Mas (FM), was assessed using the bioelectrical impedance technique using a Tanita eight-point multifrequency analyzer (Tanita BC-418 MA, Tanita Co., Tokyo, Japan). Measurements were performed under standardized conditions according to the manufacturer’s protocol: fasting for at least four hours, avoiding vigorous physical activity for at least 12 h before the study, abstaining from alcohol for 24 h and caffeine for four hours before the study, and voiding urine before BIA analysis [35]. For further analyses, the cut-off points of 30% of body fat in women and 25% in men were assumed to indicate excess body fat [36].

All measurements were performed under strictly standardized conditions (room temperature 22 °C, air humidity 45%) by one well-trained researcher (dietitian) using the same device. Measurements were performed twice in light clothing and without shoes, and averages were calculated.

### 2.5. Biochemical Analysis

In assessing the level of the lipophilic compounds, the research material was venous blood collected by qualified personnel according to the standard procedure in laboratory conditions. Blood was collected from patients after a 12-h fast, after overnight rest, from a peripheral vein in the lower elbow joint. The total cholesterol, LDL cholesterol, HDL cholesterol, triacylglycerols, and CRP as a marker of inflammation were assessed in cooperation with an external laboratory by the procedure in force at a given facility.

Based on the reference values used in the laboratory, the following cut-off points for the lipid profile were adopted and interpreted as correct levels:

- Total Cholesterol (TC) < 190 mg/dL;
- High-density lipoprotein (HDL) > 50 mg/dL for women and 40 mg/dL for men;
- Low-density Lipoprotein (LDL) < 115 mg/dL;
- Triacylglycerols (TG) < 150 mg/dL.

Additionally, blood was taken to test for vitamin E and FA. After blood was collected from the study participants, the samples were centrifuged in a rotary centrifuge for 10 min at +4 °C, 8000 rpm. The plasma from the sediment was transferred to plastic tubes. All collected plasma samples were protected from light and frozen at −80 °C until analysis.

#### 2.5.1. Plasma Vitamin E Analysis

Drawing from our study, which evaluated the intake of all eight vitamin E isoforms, four with the highest consumption levels in the Polish population were prioritized for further analysis [7]. Research indicates that typical diets predominantly provide tocopherols, particularly α-tocopherol and γ-tocopherol [37]. Consequently, the selected isoforms for detailed examination included α-tocopherol (α-T), γ-tocopherol (γ-T), α-tocotrienol (α-T3), and γ-tocotrienol (γ-T3).

High-performance liquid chromatography and a diode array detector (HPLC-DAD) were used to determine selected tocopherols and tocotrienols. In brief, 200 μL of plasma was transferred to a 2 mL Eppendorf tube and deproteinized with 400 μL of a methanol solution containing 0.04% BHT as an antioxidant. Subsequently, 800 μL of n-hexane solution with 0.04% BHT was added to the deproteinized sample and extracted for 3 min in a shaker. Following this step, the samples underwent centrifugation for 10 min in a rotary centrifuge (MPW Med. Instruments, Warsaw, Poland) at a shaking speed of 8000 rpm and a temperature of 4 °C. Post-centrifugation, 700 μL of supernatant was transferred to a new tube, followed by evaporation in a vacuum evaporator (Labconco Corporation, Kansas City, MO, USA), for 10 min at 30 °C. Then the obtained sample was reconstituted in 180 μL of methanol. The sample injection volume was 50 μL. The HPLC analysis was conducted using a specialized HPLC system (Knauer Azura, Berlin, Germany) consisting of a P6.1L pump, DAD 2.1L detector, HT310L autosampler, and CT 2.1 thermostat. An analytical column C18 Grace Vydac 201TP54 (5 μm particle size, 250 mm length × 4.60 mm) was utilized to perform the chromatographic separation. The HPLC methodology was adopted from established techniques reported by Abidi [38]. The elution was performed in isocratic mode using a 9/1, (*v*/*v*) mixture of methanol and water at the constant flow rate of 1.0 mL/min. ClarityChrom chromatographic 9.1. software was used for instrument control, data acquisition, and data processing. Detection of α-tocopherol and α-tocotrienol, γ-tocotrienols, and γ-tocopherol was performed at 292 nm, 295 nm, and 298 nm, respectively. Vitamin E compounds were identified based on the retention time values compared to standards of tocopherols and tocotrienols (LGC Standards Sp. z o.o., Kiełpin, Poland), and their concentrations were recalculated and expressed as μmol/L. Each sample underwent separate duplicate analyses, and the average of the two measurements was utilized for subsequent analysis.

Based on the plasma concentration of α-tocopherol, the nutritional status was assessed as (1) low for values below 12 μmol/L, (2) adequate/optimal for the range between 12 and 30 μmol/L interpreted, and (3) pro-healthy for values above 30 μmol/L [39].

The ratio α-tocopherol/total lipid concentration (cholesterol + triacylglycerols) was also taken into account for further analyses as a marker of vitamin E status [18].

#### 2.5.2. Plasma Fatty Acids Analysis as a Marker of Dietary Fat Intake

The plasma fatty acid (FA) composition can be used as an objective biomarker of dietary FA intake [40,41], and lipid metabolism [42].

Total plasma lipids were extracted via direct transesterification to fatty acid methyl esters (FAME), as described by Nikolic Turnic et al. [43]. Briefly, 1.5 mL of 3 M HCl in methanol was added to 100 µL of plasma, and the mixture was heated to 85 °C for 45 min. The samples were then cooled to room temperature, after which 1 mL of hexane was added. The mixture was subsequently centrifuged at 4000 rpm for 10 min at 4 °C, and the upper fraction was collected and evaporated using a CentriVap concentrator. The fatty acid profile was assessed using gas chromatography (GC) with a YL6100 GC gas chromatograph, equipped with a flame ionization detector (FID) and a BPX 70 capillary column (60 m length, 0.25 µm film thickness, and 0.25 mm internal diameter). The oven temperature was programmed as follows: 70 °C for 0.5 min, increased by 15 °C/min to 160 °C, then by 1.1 °C/min to 200 °C, held at 200 °C for 12 min, followed by a final increase of 30 °C/min to 225 °C, where it was maintained for 0.5 min. The injector and detector temperatures were set to 225 °C and 250 °C, respectively. Nitrogen was used as the carrier gas with a flow rate of 1.2 mL/min. The relative abundance of each fatty acid was quantified, and each sample was analyzed in duplicate. Results were expressed as relative percentages, determined by external normalization of the chromatographic peak areas. Fatty acids were identified by comparing the relative retention times with those of an external standard fatty acid methyl ester mixture (Supelco 37 Component FAME Mix, Sigma-Aldrich, Hamburg, Germany).

The enzymatic activity associated with fatty acid (FA) synthesis was assessed by calculating the ratios of the relative abundance of products to their respective precursors for each enzyme. Specifically, the activity of stearoyl-CoA desaturase (SCD) was evaluated using the palmitoleic-to-palmitic acid ratio (16:1*n*-7/16:0) for SCD-16, and the oleic-to-stearic acid ratio (18:1*n*-9/18:0) for SCD-18. In a similar manner, the activity of Δ6-desaturase (D6D) was determined from the dihomo-γ-linolenic-to-linoleic acid ratio (20:3*n*-6/18:2*n*-6), while Δ5-desaturase (D5D) activity was derived from the arachidonic to the dihomo-γ-linolenic acid ratio (20:4*n*-6/20:3*n*-6). Additionally, the ratio of stearic to palmitic acids (18:0/16:0) was used to estimate elongase activity [44].

### 2.6. Data Analysis

The data were reported as percentages for categorical variables and as means with standard deviations (SD) for continuous variables. Prior to conducting statistical tests, the normality of the data distribution was evaluated using the Shapiro–Wilk test. Group differences were assessed using the Pearson Chi-square test for categorical variables and either the Kruskal–Wallis test with post-hoc analysis for comparisons involving more than two groups or the Mann–Whitney U test for two-group comparisons of continuous variables. We used Spearman rank correlation analyses to investigate the link between the concentrations of vitamin E isoforms and potential determinants, including anthropometric variables, enzyme activities, fatty acid profiles, and lipid parameters. For variables showing significant correlations, we comprehensively evaluated all effects using univariate and multivariate regression analysis to identify the potential and the most robust predictors of α-tocopherol and other vitamin E isoforms. Additionally, we conducted stepwise forward multiple regression analysis to identify the potential determinants of vitamin E. The models were adjusted for age, gender and smoking status. The analyses were conducted using untransformed variables and were stratified by the total group, as well as separately for women and men. A significance threshold of *p* ≤ 0.05 was adopted for all statistical tests. The analyses were performed using STATISTICA software version 13.0 (StatSoft Inc., Tulsa, OK, USA; StatSoft, Krakow, Poland). Additionally, the sample size for each group was initially estimated to be 64 participants (128 total) using G*Power 3.1.9.7. software. The calculations were based on the assumption of medium effects (Cohen’s d = 0.5), the difference between two independent means (two groups), statistical power 0.8, and alpha significance level 0.05. Finally, for the obtained results of the main examined factor which was the percentage of body fat, the effect size was obtained in the form of Cohen’s d = 2.8 (it was correctly calculated on the basis of the difference of means and common standard deviation), and the statistical power of the test was 0.9.

## 3. Results

### 3.1. Participant Characteristics

The study included 127 adult participants, of whom 61% were women (Table 1). The mean age was 49 ± 6 years. Over 80% had at least a secondary education, 77% lived in big cities, and 67% were employed full-time. Additionally, 79% indicated non-use of tobacco products, and almost half (49%) rated their health status as good or very good. Based on body fat content (Table 1), 47% of the studied group was classified as having normal body fat levels, while 53% as excess body fat. Individuals with excess body fat content were statistically significantly older than those with normal body fat levels (52 years vs. 46 years) and more frequently reported their health status as “bad” or “not bad not good” compared to those with normal body fat content (24% and 61% vs. 2% and 12%, respectively). A higher proportion of women was classified as having excess body fat (56%) than men (44%).

### 3.2. Lipid Profile, CRP and Fatty Acids Composition

Statistically significant lowest HDL and LDL cholesterol levels were identified in the group with excess body fat (Table 2). The vast majority (92%) of individuals with normal BF met the reference value for HDL cholesterol. In contrast, in 66% of individuals with excess BF HDL cholesterol was under recommendation. Similarly, TG concentration was higher in individuals with excess BF compared with those with normal BF (64% vs. 17%).

A significantly lower inflammation, as measured by CRP concentration, was found in individuals with normal BF compared to those with excess BF (0.118 mg/dL for women and 0.805 mg/dL for men vs. 1.8 mg/dL and 3.4 mg/dL, respectively) (Table 2).

Individuals with excess BF showed different plasma FA profiles. Significantly higher of MUFA (30.5% for women and 33.8% for men vs. 22.7% and 25.8%, respectively), lower PUFA (32.2% for women and 31.1% for men vs. 37.6% and 37.0%, respectively), lower of *n*-3 (5.18% for women and 4.46% for men vs. 4.01% and 3.71%, respectively), and *n*-6 (27.1% for women and 26.7% for men vs. 33.6% and 33.3%, respectively), compared to individuals with excess BF.

### 3.3. Vitamin E Status and Isoforms in Dependence of Body Fat Content

Table 3 presents the results regarding plasma levels of vitamin E isoforms (α-tocopherol, γ-tocopherol, α-tocotrienol, γ-tocotrienol) and their total concentration by BF content (normal vs. excess). A low nutritional status of vitamin E was observed in 21% of the study participants, while a health-promoting status was identified in 30%. Individuals with a normal BF exhibited significantly higher concentrations of all examined vitamin E isoforms than individuals with excess BF (32.9 vs. 19,6 µmol/L for α-tocopherol, 12.6 vs. 8.69 µmol/L for γ-tocopherol, 7.79 vs. 4.41 µmol/L for α-tocotrienol, 10.7 vs. 7.47 µmol/L for γ-tocotrienol and 64.0 vs. 40.2 µmol/L for sum of Ts and T3s, *p* < 0.001). A pro-healthy status of α-T (>30 µmol/L) was observed in 50% of individuals with normal BF content and in 12% with excess BF. Individuals with excess BF were significantly more likely to exhibit a low nutritional status of vitamin E (30% vs. 12%) and less likely to display a health-promoting status (12% vs. 50%) compared to individuals with normal BF.

### 3.4. Association Between Plasma Ts and T3s Concentration and Anthropometrics, Plasma Fatty Acids, Lipid Profile, Enzymes Activity and Inflammation

Negative correlations (Table 4) were observed between plasma α-T concentration and WHtR (r = −0.394), FM (r = −0.387), WC (r = −0.382), BF (r = −0.359), BMI (r = −0.346), BW (r = −0.340), HC (r = −0.334), and WHR (r = −0.277). Similar patterns were identified for other isoforms of vitamin E (α- and γ-) and their combined total.

The level of sum Ts and T3s showed a negative correlation with the sum of MUFA (r = −0.339), but a positive correlation with PUFA (r = 0.242).

A positive correlation was observed between α-T and HDL (r = 0.487) as well as LDL (r = 0.255), while a negative correlation was noted with TG (r = −0.295). Similar trends were seen for other isoforms of vitamin E (α- and γ-) and their combined total. However, TC and LDL were not significant for the other vitamin E forms. All vitamin E isoforms had the strongest correlation with HDL. Moreover, the strongest correlation was found between α-T/TL and TG (r = −0.625).

The concentration of all vitamin E isoforms strongly correlated negatively with CRP levels. Although the correlations for γ- isoforms were weaker, but still negative.

Significant negative correlations were also identified for SCD-16 with various vitamin E isoforms (except γ-tocotrienol), their combined total, and lipid-adjusted α-tocopherol concentration (e.g., r = −0.265 for α-T, r = −0.247 for α-T3, r = −0.194 for γ-T, r = −0.235 for the sum of tocopherols and tocotrienols, and r = −0.377 for α-T/TL). SCD-18 exhibited similar trends, albeit with slightly weaker correlations, showing negative relationships with α-T (r = −0.237), α-T3 (r = −0.197), γ-T (r = −0.182), the sum of tocopherols and tocotrienols (r = −0.236), and α-T/TL (r = −0.232). Furthermore, D6D negatively correlated with lipid-adjusted α-tocopherol concentration (r = −0.234). In contrast, no significant associations were observed between vitamin E levels and the activities of D5D or Elongase.

Univariate linear regression (Table 5) showed that significant predictors for higher vitamin E isoform concentration were higher HDL, lower: MUFA, CRP, WC, WHtR, FM and BMI. Stronger associations were found for α- forms.

Results of multivariate linear regression analysis (Table 6) revealed that the most significant predictors of α-T were the sum of MUFA (β = 0.476, 95% CI: −0.933; −0.019, *p* = 0.041) and HDL (β = 0.371, 95% CI: 0.071; 0.671, *p* = 0.016). This model explained 29% of the variance in α-T concentration. The most significant predictors for α-T3 were HDL (β = 0.347, 95% CI: 0.011; 0.684, *p* = 0.043). This model explained 22% of the variance in α-T3 concentration. For γ-Tand for γ-T3, HDL was a significant predictor (β = 0.330, 95% CI: 0.014; 0.646, *p* = 0.041 and β = 0.364, 95% CI: 0.023; 0.705, *p* =0.036); however, the overall models were not statistically significant (R^2^ = 0.017, *p* = 0.118 and R^2^ = 0.172, *p* = 0.105).

Since previous results revealed significant predictors for α-tocopherol, and considering that this form of vitamin E is recognized as the primary biologically active form, further analyses were conducted using stepwise multiple linear regression. This approach aimed to determine whether the automatic elimination of variables would uncover new associations. Model 1, without adjustments, indicates (Table 7) that plasma α-tocopherol concentration is associated with decreased BF (β = −0.201, 95% CI: −0.389; −0.012, *p* = 0.037). Additionally, α-tocopherol concentration is positively associated with TC (β = 0.234, 95% CI: 0.073; 0.394, *p* = 0.005) and HDL cholesterol (β = 0.230, 95% CI: 0.038; 0.421, *p* = 0.019). After adjustment for age, sex, and smoking status (model 2), an increase in plasma α-tocopherol concentration is associated with higher HDL cholesterol (β = 0.389, 95% CI: 0.201; 0.578, *p* < 0.001) and LDL cholesterol (β = 0.231, 95% CI: 0.068; 0.395, *p* = 0.006). However, in women, only HDL cholesterol was significantly associated with the α-tocopherol concentration (β = 0.443, 95% CI: 0.203; 0.684, *p* < 0.001), whereas in men, a significant association was observed with LDL cholesterol (β = 0.429, 95% CI: 0.148; 0.800, *p* = 0.004).

## 4. Discussion

The low nutrition status of vitamin E measured by α-tocopherol plasma concentrations was found in 27% of our group. We found that the determinants of lower plasma vitamin E were higher BF and lower TC and its fraction, with the strongest correlations being found for HDL. This partially confirmed our hypothesis that a lower concentration of vitamin E isoforms is linked with poorer metabolic health. Vitamin E isoform concentration was negatively correlated with CRP, but in-depth analyses did not confirm this association. Our findings indicated the need for further studies to explain these associations.

In our study, 12% of participants with normal body fat and 30% of participants with excess body fat had low plasma α-tocopherol concentrations. Only 30% of participants had pro-healthy plasma status vitamin E, and only 12% of those with excess body fat. The average plasma vitamin E concentrations in our research were comparable to those of other studies [45], or slightly higher [46,47]. This is consistent with the results of Peter et al. [39], who found that α-tocopherol status was inadequate in a significant part of the studied populations, with only 21% globally reaching a plasma concentration of ≥30 µmol/L, which is considered beneficial for health.

Among subjects with excess body fat, we observed significantly lower plasma concentrations of all vitamin E isoforms compared to healthy controls, both in terms of mean concentrations and the frequency of optimal levels. Participants with lower vitamin E concentrations also exhibited higher values of anthropometric indices, such as body weight, waist circumference, BMI, WHR, and WHtR. Other studies have reported similarly low levels of vitamin E in overweight or obese individuals [48,49].

Additionally, research involving postmenopausal women showed a strong positive correlation between α-tocopherol and waist-to-hip ratio, with no significant associations observed with waist circumference or BMI. This may indicate that α-tocopherol is more closely associated with fat distribution than total body fat [50]. However, Meulmeester et al. [51] found no significant relationship between adiposity measures and plasma α-tocopherol levels in middle-aged individuals, though they report that higher BMI and total body fat were associated with lower levels of oxidized α-tocopherol metabolites, suggesting reduced antioxidant activity in individuals with increased abdominal fat. Although no overall differences in vitamin E isoform concentrations were observed between men and women, gender-specific patterns emerged. Women with excess body fat exhibited significantly lower concentrations of all four tested vitamin E isoforms compared to women with normal body fat. In contrast, significant differences in vitamin E levels were observed only in α-tocopherol and tocotrienol concentrations among men. These findings suggest that body fat may influence vitamin E metabolism differently in men and women, potentially due to variations in fat distribution and metabolic pathways.

Gender-specific differences in vitamin E metabolism are influenced by hormonal factors, particularly estrogen and testosterone, which regulate fat distribution and lipid metabolism. These hormones play a pivotal role in storing, mobilizing, and utilizing vitamin E in men and women. Estrogen promotes subcutaneous fat accumulation, which is more prevalent in women, potentially affecting the storage and release of vitamin E. Testosterone, on the other hand, is associated with visceral fat accumulation in men, influencing lipid metabolism and vitamin E utilization [52]. Sex hormones also differentially regulate lipid metabolism genes in male and female hepatocytes, impacting vitamin E metabolism. For instance, 17β-estradiol modulates the expression of genes involved in lipid metabolism in female hepatocytes, whereas testosterone influences distinct gene pathways in males. Recognizing these sex-based metabolic differences is crucial for developing effective vitamin E supplementation strategies. Tailoring dosages or delivery methods based on individual fat distribution patterns and hormonal profiles may enhance the outcomes of nutritional interventions [53].

Nutritional guidelines for vitamin E intake differ by gender, which could potentially affect plasma vitamin E levels [54]. Additionally, differences in body fat composition between men and women may also play a significant role in vitamin E distribution and metabolism [55]. Despite these differences, optimal plasma vitamin E concentrations are generally consistent for both genders, making it essential to explore whether gender impacts these levels [54]. Furthermore, age groups need to be taken into account to verify current recommendations. Studies have shown that one of the main functions of vitamin E is to correct impaired immune and inflammatory cell functions in older adults, which has not been considered in recommendations to date [16]. However, it is well documented that older adults (>65 years) have impaired immune and inflammatory responses, as well as enzymatic antioxidant defense mechanisms, which increase the risk of chronic infectious and noncommunicable diseases compared to younger adults [56]. The aging process is associated with a dysregulation of immune function, a phenomenon often referred to as “immunosenescence,” characterized by hyporesponsive cellular immune responses and pathogen defenses, coupled with a prolonged inflammatory state known as “inflammaging” [16]. Among the issues to consider is the efficacy of other forms of vitamin E. In this context, there is emerging evidence that other vitamin E homologues are unique and may potentially have higher efficacy. Based on the current state of research, γ-T and T3, as well as the hepatic metabolites α- and γ-T, are promising forms of vitamin E in the prevention of diseases caused by acute inflammatory and oxidative processes [16,56].

Our findings confirmed that plasma concentrations of total cholesterol, HDL, and LDL were the main predictors of vitamin E concentration, and HDL cholesterol played a pivotal role across all vitamin E isoforms [57,58]. Interestingly, individuals with normal body fat exhibit significantly higher HDL and LDL levels compared to those with excess body fat, which may further explain elevated vitamin E levels. Despite this, the literature presents divergent findings regarding the direction and strength of lipid vitamin E correlations [57,58]. Nevertheless, HDL is consistently identified as positively associated with vitamin E concentrations. Given that dyslipidemia, characterized by high TG and LDL levels, and low HDL levels, is a primary contributor to cardiovascular disease (CVD) [58] this relationship is crucial. Dyslipidemia is strongly linked to obesity, non-alcoholic fatty liver disease (NAFLD), and other metabolic disorders [59]. Vitamin E, a crucial antioxidant, has been shown to mitigate CVD risk through mechanisms such as inhibiting LDL cholesterol oxidation and reducing oxidative stress in NAFLD [60]. Given the strong association between dyslipidemia, obesity, and oxidative stress, vitamin E supplementation is particularly recommended for individuals with elevated LDL levels and obesity. Studies suggest that vitamin E may prevent LDL particle oxidation, a critical factor in atherosclerosis progression [61]. Consequently, its role in CVD prevention, particularly in populations with metabolic disorders, confirmed the potential of vitamin E as a complementary therapeutic agent.

Our study found that participants with higher adiposity exhibited significantly elevated levels of monounsaturated fatty acids (MUFA) and omega-3 fatty acids, whereas individuals with normal body fat demonstrated higher concentrations of polyunsaturated fatty acids (PUFA), particularly omega-6 fatty acids. This divergence in fatty acid composition suggests an underlying disruption in lipid metabolism associated with obesity, which may influence both metabolic health and oxidative stress levels [62]. Additionally, fatty acids serve as potential dietary intake markers, reflecting the consumption of specific fats in the diet. The observed variations in fatty acid profiles across different body fat levels may indicate distinct dietary patterns or metabolic responses to fat intake. Elevated MUFA and omega-3 levels in individuals with excess body fat could be a consequence of altered lipid processing or increased dietary fat intake. In contrast, the higher PUFA and omega-6 levels in individuals with normal body fat may be indicative of a diet richer in these essential fatty acids, which are known to play a critical role in cellular integrity and metabolic regulation [63,64].

We also observed that individuals with adiposity had exhibited elevated activities of some enzyme activity involved in fatty acid metabolism. The activity of Δ6-desaturase, stearoyl-CoA desaturase-16, and stearoyl-CoA desaturase-18 was higher, whereas Δ5-desaturase (D5D) was more active in those with normal body weight. Increased D6D activity may enhance pro-inflammatory eicosanoid synthesis, commonly linked to chronic inflammation in obesity [65]. Elevated stearoyl-CoA desaturase (SCD) activity significantly influences vitamin E concentrations and its antioxidant function through its role in lipid metabolism [66]. SCD catalyzes the conversion of SFAs into MUFAs, such as oleic acid, which are key components of triglycerides, phospholipids, and cholesterol esters that transport and store vitamin E [67]. Changes in SCD activity can alter the lipid microenvironment, potentially impacting vitamin E bioavailability and efficacy. MUFAs are less susceptible to peroxidation than polyunsaturated fatty acids, reducing the oxidative demand on vitamin E and potentially modifying its antioxidant requirements [68]. However, elevated SCD activity is often linked to lipid accumulation in tissues, which may alter local vitamin E concentrations and impair its protective role. This lipid accumulation is associated with insulin resistance and metabolic disturbances, as MUFAs contribute to triacylglycerol synthesis, potentially leading to fat deposition in the liver and peripheral tissues [68]. Conversely, D5D converts dihomo-γ-linolenic acid (DGLA) into anti-inflammatory PUFAs, suggesting that higher D5D activity in normal-weight individuals supports a healthier lipid profile and metabolic health. However, in our study, vitamin E isoforms negatively correlated with SCD-16 and SCD-18 activity, but their association was not confirmed by multivariate linear regression, which requires further study.

Our study demonstrates inflammation was linked with excess body fat. Vitamin E isoforms showed significant negative correlations with CRP, and confirmed a potential anti-inflammatory role for vitamin E. Similarly, Mazidi et al. [69] found that higher antioxidant levels, including vitamin E, were associated with lower CRP concentrations, with obesity moderately influencing this relationship. Moreover, results of meta-analyses indicated that α-tocopherol supplementation can reduce CRP by 0.52 to 0.62 mg/L, underscoring its anti-inflammatory properties [4]. Furthermore, observational meta-analyses report that over half (17/27) of randomized controlled trials (RCTs) identified statistically significant protective associations between vitamin E and various health parameters, including CRP, further supporting its role in inflammation reduction [70].

Several limitations of our study should be acknowledged. Firstly, we did not collect dietary data to assess the intake of different vitamin E isoforms and fatty acids. According to other studies, we assumed that their consumption is related to the consumption of plant fats, for which the plasma fatty acid profile is a better biomarker. Secondly, our findings concern people aged 40–60 years and do not apply to other age groups. Additionally, more precise biomarkers of vitamin E status, such as α-tocopherol concentrations in adipose tissue obtained through biopsies or vitamin E metabolites, should be considered for future research, as plasma α-tocopherol levels alone may not fully reflect long-term vitamin E status. Nutritional status of vitamin E is affected by numerous factors, e.g., dietary intake of vitamin E, other antioxidant and pro-oxidant compounds, absorption efficiency, and vitamin E catabolism, but bioavailability has been shown to be its key determinant. Significant variability in plasma vitamin E isoform levels among participants was observed, potentially stemming from differences in individual absorption, metabolism, or dietary habits that were not accounted for. We recognize that genetic variation, including single nucleotide polymorphisms associated with fasting blood vitamin E concentration and α-tocopherol bioavailability, could have influenced plasma vitamin E levels [71]. The interindividual variability in α-tocopherol bioavailability is associated with a combination of 28 SNPs in or near 11 candidate genes. Four of these genes SLC10A2 (solute carrier family 10 (sodium/bile acid cotransporter), member 2), PNLIP (pancreatic lipase), SREBF2 (sterol regulatory element binding transcription factor 2), and ABCG1 (ATP-binding cassette, subfamily G (WHITE), member 1) play a specific role in α-tocopherol bioavailability [71]. This variability could affect the generalizability of the findings, as outcomes may differ across diverse populations or settings. Furthermore, the observational nature of the study limits causal inferences between α-tocopherol levels and metabolic health. While correlations were identified, they do not confirm causality, as various confounding factors—such as diet, genetic predispositions, and health behaviors—may have influenced the results. Although C-reactive protein (CRP) is a well-established marker of inflammation, the study could benefit from incorporating more sensitive and specific biomarkers for detecting inflammatory or metabolic conditions. Additionally, the potential influence of other nutrients on the function and status of vitamin E was not assessed in this study. The synergistic antioxidant effects and mutual regeneration capacity of vitamins C and E are particularly significant, as they are crucial in maintaining oxidative balance within the body [72]. Additionally, the combined antioxidant activity of vitamin E with carotenoids, and also selenium, and zinc as cofactors of antioxidant enzymes further underscores the importance of nutrient interactions in modulating oxidative stress [73]. Moreover, the interplay between vitamins E and K warrants attention, as vitamin E may interfere with vitamin K’s essential role in blood coagulation [74]. Including such parameters in future research could provide a more comprehensive understanding of the interplay between antioxidants and metabolic health.

Indices of central obesity, such as waist circumference (WC) and waist-to-height ratio (WHtR), did not reveal significant associations with vitamin E status in our study. While these measures are commonly used to estimate the central fat distribution and associated metabolic risks, their inability to differentiate between visceral and subcutaneous fat might limit their predictive value for specific outcomes like vitamin E metabolism. Advanced imaging techniques, such as DEXA, MRI, or CT scans, could provide a more precise assessment of visceral and subcutaneous fat depots. The ability to distinguish between these types of fat could enhance our understanding of their specific roles in vitamin E metabolism and health outcomes.

The undoubted strength of this study is the comprehensive assessment of multiple lipophilic components related to vitamin E metabolism. Moreover, the calculated effect size allows us to assume that the sample size was sufficient to statistically confirm the obtained relationships. In our research, more isoforms of vitamin E were analyzed, extending beyond alpha-tocopherol, to provide a more comprehensive assessment of the nutritional status of vitamin E.

## 5. Conclusions

In our study, 12% of participants with normal body fat and 30% of participants with excess body fat had low plasma α-tocopherol nutritional status. Only 30% of participants had a pro-healthy plasma status of vitamin E, and only 12% of those with excess body fat. These results found that the vitamin E status of adults with excess body fat may be inadequate and linked to poorer metabolic health. We found that the determinants of lower plasma vitamin E were higher BF and lower TC and its fraction, with the strongest correlations being found for HDL. This partially confirmed our hypothesis that a lower concentration of vitamin E isoforms is linked with poorer metabolic health. Considering that the risk of metabolic diseases increases with age and with adiposity, but is also associated with a biological change in body composition towards increasing fat mass, the pro-healthy nutritional status of vitamin E through would be beneficial for health.

## Figures and Tables

**Table 1:** Baseline characteristics of the study subjects and according to body fat content.

Variables	Total (n = 127)	Body Fat Content (%)		*p*-Value
		Normal (n = 60)	Excess (n = 67)	
Sociodemographic (n, %)				
Women	77 (61)	34 (57) 26 (43)	43 (64)	NS
Men	50 (39)		24 (36)	
Age in years (mean ± SD)	49 ± 6	46 ± 5	52 ± 6	<0.001
Education (n, %)				
Primary and basic vocational	15 (12)	3 (5)	12 (18)	<0.001
Secondary	42 (33)	7 (12)	35 (52)	
University	70 (55)	50 (83)	20 (30)	
Place of living (n, %)				
Village	10 (8)	3 (5)	7 (10)	NS
City < 100,000 inhab.	19 (15)	5 (8)	14 (21)	
City > 100,000 inhab.	98 (77)	52 (87)	46 (69)	
Professional status (n, %)				
Not working	24 (19)	6 (10)	18 (27)	NS
Work part-time	18 (14)	10 (17)	8 (12)	
Work full time	85 (67)	44 (73)	41 (61)	
Smoking (n, %)				
Yes	27 (21)	13 (22)	14 (21)	NS
No	100 (79)	47 (78)	53 (79)	
Health status self-assessment (n, %)				
Bad	17 (13)	1 (2)	16 (24)	<0.001
Not bad not good	48 (38)	7 (12)	41 (61)	
Good or very good	62 (49)	52 (87)	10 (15)	

NS, not significant; results of the *t*-Student test or the U-Mann–Whitney test; *p*-value < 0.05.

**Table 2:** Lipid profile and fatty acid composition and body fat content.

		Body Fat Content (%)						
Variables	Total (n = 127)	Normal (n = 60)		*p*-Value	Excess (n = 67)		*p*-Value	*p*-Value
		Women (n = 34)	Men (n = 26)		Women (n = 43)	Men (n = 24)		Normal vs. Excess
			Lipid profile					
TC (mg/dL)	199.7 ± 33.4	197.3 ± 28.2	204.5 ± 37.1	NS	201.3 ± 34.7	194.8 ± 34.7	NS	NS
<190 mg/dL, n (%)		13 (38)	11 (42)	NS	12 (28)	12 (50)	NS	NS
≥190 mg/dL, n (%)		21(62)	15 (58)		31 (43)	12 (50)		
HDL (mg/dL)	53.2 ± 16.8	71.2 ± 10.1	54.8 ± 14.5	<0.001	46.2 ± 11.0	38.6 ± 12.6	0.002	<0.001
>50 mg/dL for women and 40 mg/dL for men, n (%)	78 (61)	33 (97)	22 (85)	NS	15 (35)	8 (33)	NS	<0.001
≤50 mg/dL for women and 40 mg/dL for men, n (%)	49 (39)	1 (3)	4 (15)		28 (65)	16 (67)		
LDL (mg/dL)	118.2 ± 32.0	121.5 ± 28.5	132.2 ± 35.2	NS	113.1 ± 30.8	107.4 ± 31.0	NS	<0.001
<115 mg/dL, n (%)	61 (48)	14 (41)	10 (38)	NS	22 (51)	15 (63)	NS	NS
≥115 mg/dL, n (%)	66 (52)	20 (59)	16 (62)		21 (49)	9 (37)		
TG (mg/dL)	139.3 ± 78.6	77.7 ± 35.0	118.0 ± 81.1	NS	168.5 ± 55.0	197.4 ± 91.2	NS	<0.001
<150 mg/dL, n (%)	74 (58)	31 (91)	19 (73)	NS	16 (37)	8 (33)	NS	<0.001
≥150 mg/dL, n (%)	53 (42)	3 (9)	7 (27)		27 (63)	16 (67)		
			Inflammation					
CRP (mg/dL)	1.5 ± 3.4	0.118 ± 0.055	0.805 ± 2.5	0.034	1.8 ± 2.6	3.401 ± 6.03	NS	<0.001
	Plasma fatty acids (results for selected FA)							
Σ SFA (%)	37.5 ± 7.5	39.6 ± 10.3	37.2 ± 5.5	NS	37.3 ± 7.1	35.1 ± 4.5	NS	NS
C 15:0	0.82 ± 3.7	1.04 ± 4.9	0.23 ± 0.1	NS	1.32 ± 4.7	0.26 ± 0.1	NS	0.024
C 16:0	21.8 ± 5.5	20.8 ± 5.3	22.8 ± 3.2	NS	21.5 ± 7.0	22.6 ± 4.8	NS	NS
C 17:0	0.26 ± 0.2	0.16 ± 0.2	0.21 ± 0.1	NS	0.33 ± 0.3	0.30 ± 0.2	NS	<0.001
C 18:0	9.5 ± 2.8	9.75 ± 1.7	8.65 ± 1.5	0.011	10.3 ± 4.1	8.67 ± 1.8	0.028	NS
Σ MUFA (%)	28.1 ± 6.6	22.7 ± 4.6	25.8 ± 5.7	0.025	30.5 ± 5.1	33.8 ± 5.4	0.015	<0.001
C 16:1	1.89 ± 1.0	1.22 ± 0.75	1.65 ± 0.8	0.037	2.28 ± 1.0	2.39 ± 1.0	NS	<0.001
C 18:1 *n*-9	25.8 ± 5.9	21.4 ± 4.25	24.1 ± 5.1	0.033	27.4 ± 5.2	31.0 ± 4.9	0.007	<0.001
Σ PUFA (%)	34.4 ± 7.3	37.6 ± 8.4	37.0 ± 6.4	NS	32.2 ± 6.5	31.1 ± 5.2	NS	<0.001
Σ *n*-3 (%)	4.43 ± 2.4	4.01 ± 2.4	3.71 ± 1.8	NS	5.18 ± 2.6	4.46 ± 2.0	NS	0.005
C 18:3 *n*-3	0.61 ± 0.4	0.482 ± 0.4	0.52 ± 0.3	NS	0.66 ± 0.3	0.82 ± 0.5	NS	0.0004
C 20:5 *n*-3	1.01 ± 1.0	0.958 ± 0.9	0.75 ± 0.7	NS	1.16 ± 1.0	1.14 ± 1.3	NS	0.029
C 22:6 *n*-3	2.80 ± 1.6	2.573 ± 1.7	2.44 ± 1.3	NS	3.37 ± 1.8	2.51 ± 1.1	0.039	0.039
Σ *n*-6 (%)	30.0 ± 6.8	33.6 ± 7.7	33.3 ± 6.1	NS	27.1 ± 5.4	26.7 ± 4.3	NS	<0.001
C 18:2 *n*-6	21.4 ± 7.1	25.7 ± 7.9	24.8 ± 6.6	NS	17.7 ± 4.8	18.4 ± 5.0	NS	<0.001
C 18:3 *n*-6	0.49 ± 1.8	0.210 ± 0.2	0.29 ± 0.2	NS	0.49 ± 0.6	1.14 ± 4.0	0.016	0.006
C 20:3 *n*-6	2.32 ± 1.5	1.66 ± 1.1	2.31 ± 1.5	NS	2.99 ± 1.6	2.06 ± 1.4	0.021	0.009
C 20:4 *n*-6	5.76 ± 1.7	6.02 ± 1.9	5.84 ± 2.3	NS	5.89 ± 1.5	5.05 ± 1.0	0.005	NS
*n*-3/*n*-6	0.154 ± 0.09	0.121 ± 0.08	0.115 ± 0.06	NS	0.197 ± 0.1	0.168 ± 0.07	NS	<0.001

NS, not significant; TC, total cholesterol; LDL, low-density lipoprotein; HDL, high-density lipoprotein; TG, triacylglycerol; CRP, C-reactive protein, FA, fatty acids; Σ, sum; SFA, saturated fatty acids; MUFA, monounsaturated fatty acids; PUFA, polyunsaturated fatty acids; *n*-3, omega 3; *n*-6, omega6; results of the Chi^2^ or *t*-Student test or the U-Mann–Whitney test; *p*-value < 0.05.

**Table 3:** Plasma Vitamin E isoforms and assessment of vitamin status and body fat content.

		Body Fat Content (%)		
Variables	Total (n = 127)	Normal (n = 60)	Excess (n = 67)	*p*-Value
α—T (µmol/L)	25.9 ± 17.6	32.9 ± 19.5	19.6 ± 12.9	<0.001
Low, n (%)	27 (21)	7 (12)	20 (30)	<0.001
Adequate, n (%)	62 (49)	23 (38)	39 (58)	
Pro-Healthy, n (%)	38 (30)	30 (50)	8 (12)	
γ—T (µmol/L)	10.5 ± 9.1	12.6 ± 9.5	8.69 ± 8.3	<0.001
α—T3 (µmol/L)	6.0 ± 4.5	7.79 ± 4.1	4.41 ± 4.2	<0.001
γ—T3 (µmol/L)	9.0 ± 6.8	10.7 ± 5.7	7.47 ± 7.4	<0.001
Sum of Ts and T3s (µmol/L)	51.5 ± 31.4	64.0 ± 31.4	40.2 ± 26.9	<0.001

T, tocopherol; T3, tocotrienol; results of the Chi^2^ or *t*-Student test or the U-Mann–Whitney test; *p*-value < 0.05.

**Table 4:** Correlation coefficients of anthropometric variables, fatty acids, enzyme activity and lipid profile in different vitamin E isoforms.

Variables	α-T	α-T3	γ-T	γ-T3	Sum of Ts and T3s	α-T/TL
BW (kg)	−0.340	−0.237	−0.219	-	−0.296	-
WC (cm)	−0.382	−0.287	−0.231	−0.227	−0.343	−0.241
HC (cm)	−0.334	−0.226	-	-	−0.279	−0.327
BF (%)	−0.359	−0.333	-	−0.276	−0.348	−0.584
FM (kg)	−0.387	−0.333	−0.230	−0.261	−0.365	−0.416
BMI (kg/m^2^)	−0.346	−0.288	-	−0.228	−0.321	−0.289
WHR	−0.277	−0.233	−0.203	−0.183	−0.259	-
WHtR	−0.394	−0.339	−0.211	−0.291	−0.381	−0.408
Σ SFA (%)	0.192	-	-	-	-	-
Σ MUFA (%)	−0.376	−0.294	−0.227	−0.238	−0.339	−0.384
Σ PUFA (%)	0.184	0.247	0.294	0.276	0.242	0.254
TC (mg/dL)	-	-	-	-	-	−0.396
HDL (mg/dL)	0.487	0.393	0.331	0.349	0.459	0.232
LDL (mg/dL)	0.255	-	-	-	-	-
TG (mg/dL)	−0.295	−0.337	−0.192	−0.268	−0.307	−0.625
CRP (mg/dL)	−0.464	−0.453	−0.270	−0.355	−0.454	−0.373
D5D	-	-	-	-	-	-
D6D	-	-	-	-	-	−0.234
SCD16	−0.265	−0.247	−0.194	-	−0.235	−0.377
SCD18	−0.237	−0.197	−0.182	-	−0.236	−0.232
Elongase	-	-	-	-	-	-

T, tocopherol; T3, tocotrienol; TL, total lipids; BW, body weight (kg), WC, waist circumference (cm), HC, hip circumference (kg), BF, body fat content (%); FM, fat mass (kg); BMI, body mass index (kg/m^2^), WHR, waist–hip ratio; WHtR, waist to height ratio; Σ, sum; SFA, saturated fatty acids; MUFA, monounsaturated fatty acids; PUFA, polyunsaturated fatty acids; TC, total cholesterol; LDL, low-density lipoprotein; HDL, high-density lipoprotein; TG, triacylglycerol; CRP, C-reactive protein; D5D, delta-5-desaturase; D6D, delta-6-desaturase; SCD16, stearoyl-CoA desaturase 16; SCD18, stearoyl-CoA desaturase; -, the result is statistically insignificant; results of the Spearman rank correlations, *p* < 0.05.

**Table 5:** Results of univariate linear regression between vitamin E isoforms and significant variables.

Variables	α-T	α-T3	γ-T	γ-T3
	β, *p*-Value	β, *p*-Value	β, *p*-Value	β, *p*-Value
BW (kg)	−0.258, 0.003	−0.249, 0.005	−0.242, 0.006	−0.130, 0.144
WC (cm)	−0.318, <0.001	−0.312, <0.001	−0.243, 0.006	−0.205, 0.021
HC (cm)	−0.292, 0.001	−0.191, 0.031	-	−0.106, 0.236
FM (kg)	−0.347, <0.001	−0.303, <0.001	−0.223, 0.012	−0.192, 0.030
BF (%)	−0.336, <0.001	−0.292, <0.001	-	−0.202, 0.023
BMI (kg/m^2^)	−0.315, <0.001	−0.290, <0.001	−0.204, 0.022	−0.176, 0.048
WHR	−0.223, 0.012	−0.302, <0.001	−0.203, 0.022	−0.213, 0.016
WHtR	−0.339, 0.001	−0.324, <0.001	−0.211, 0.017	−0.225, 0.011
Σ SFA (%)	0.069, 0.442	-	0.0006, 0.99	0.011, 0.905
Σ MUFA (%)	−0.239, 0.007	−0.322, <0.001	−0.250, 0.005	−0.234, 0.008
Σ PUFA (%)	0.143, 0.108	0.179, 0.044	0.223, 0.012	0.199, 0.024
TC (mg/dL)	0.283, <0.001	−0.033, 0.712	0.071, 0.431	−0.104, 0.245
HDL (mg/dL)	0.381, <0.001	0.383, <0.001	0.331, <0.001	0.294, 0.001
LDL (mg/dL)	0.320, <0.001	-	0.135, 0.129	−0.086, 0.335
TG (mg/dL)	−0.114, 0.201	−0.271, 0.002	−0.153, 0.085	−0.212, 0.017
CRP	−0.254, 0.004	−0.230, 0.009	−0.148, 0.097	−0.113, 0.206
D5D	-	-	−0.084, 0.345	−0.059, 0.513
D6D	-	-	0.047, 0.602	0.036, 0.689
SCD16	−0.127, 0.153	−0.126, 0.157	−0.143, 0.108	−0.004, 0.964
SCD18	−0.066, 0.463	−0.186, 0.036	−0.137, 0.124	−0.154, 0.084
Elongase	-	-	−0.030, 0.734	0.077, 0.388

T, tocopherol; T3, tocotrienol; BW, body weight (kg), WC, waist circumference (cm), HC, hip circumference (kg), BF, body fat content (%); FM, fat mass (kg); BMI, body mass index (kg/m^2^), WHR, waist–hip ratio; WHtR, waist to height ratio; Σ, sum; SFA, saturated fatty acids; MUFA, monounsaturated fatty acids; PUFA, polyunsaturated fatty acids; TC, total cholesterol; LDL, low-density lipoprotein; HDL, high-density lipoprotein; TG, triacylglycerol; CRP, C-reactive protein; D5D, delta-5-desaturase; D6D, delta-6-desaturase; SCD16, stearoyl-CoA desaturase 16; SCD18, stearoyl-CoA desaturase; -, variables not included in the analyses; Adjusted for gender, age and smoking.

**Table 6:** Multivariate linear regression between plasma vitamin E isoform concentration and selected predictors (all effects).

Variables	α-T	α-T3	γ-T	γ-T3
	β (95% CI), *p*-Value	β (95% CI), *p*-Value	β (95% CI), *p*-Value	β (95% CI), *p*-Value
Σ SFA (%)	−0.068 (−0.298; 0.162), 0.560	-	-	-
Σ MUFA (%)	−0.476 (−0.933; −0.019), 0.041	−0.333 (−0.744; 0.079), 0.112	−0.383 (−0.800; 0.035), 0.072	−0.097 (−0.367; 0.173), 0.476
Σ PUFA (%)	-	0.053 (−0.178; 0.285), 0.648	0.138 (−0.088; 0.364), 0.230	0.109 (−0.109; 0.328), 0.324
SCD16	0.164 (−0.062; 0.389), 0.154	0.084 (−0.152; 0.320), 0.481	0.071 (−0.163; 0.305), 0.549	-
SCD18	0.318 (−0.035; 0.672), 0.077	0.226 (−0.142; 0.593), 0.226	0.227 (−0.142; 0.597), 0.225	-
BW (kg)	1.182 (−1.996; 4.360), 0.462	1.316 (−1.999; 4.632), 0.433	0.460 (−0.418; 1.339), 0.301	-
WC (cm)	−0.887 (−7.656; 5.882), 0.796	−0.422 (−7.451; 6.606), 0.905	−1.367 (−3.037; 0.304), 0.108	0.629 (−0.997; 2.255), 0.445
HC (cm)	−0.742 (−2.595; 1.111), 0.429	−0.696 (−2.629; 1.237), 0.477	-	-
FM (kg)	−0.390 (−1.872; 1.092), 0.603	−0.523 (−2.069; 1.022), 0.504	−0.148 (−0.883; 0.587), 0.691	−0.509 (−2.057; 1.039), 0.516
BF (%)	0.213 (−0.695; 1.121), 0.642	0.174 (−0.773; 1.122), 0.716	-	0.278 (−0.633; 1.189), 0.546
BMI (kg/m^2^)	−0.471 (−3.665; 2.724), 0.771	−0.799 (−4.133; 2.534), 0.635	-	0.484 (−0.651; 1.619), 0.340
WHR	−0.618 (−2.757; 1.521), 0.568	−0.959 (−3.188; 1.269), 0.395	0.185 (−0.259; 0.629), 0.410	−0.122 (−0.565; 0.320), 0.583
WHtR	1.511 (−3.802; 6.824), 0.574	1.717 (−3.808; 7.244), 0.539	1.058 (−0.106; 2.221), 0.074	−0.613 (−2.249; 1.023), 0.459
CRP (mg/dL)	−0.110 (−0.305; 0.084), 0.263	−0.131 (−0.333; 0.071), 0.201	−0.059 (−0.264; 0.145), 0.565	−0.071 (−0.272; 0.131), 0.487
TC (mg/dL)	0.020 (−0.377; 0.417), 0.921	−0.015 (−0.364; 0.070), 0.182	−0.085 (−0.307; 0.136), 0.445	−0.065 (−0.284; 0.154), 0.559
HDL (mg/dL)	0.371 (0.071; 0.671), 0.016	0.347 (0.011; 0.684), 0.043	0.330 (0.014; 0.646), 0.041	0.364 (0.023; 0.705), 0.036
LDL (mg/dL)	0.166 (−0.016; 0.347), 0.413	-	-	-
TG (mg/dL)	0.181 (−0.063; 0.425), 0.144	−0.071 (−0.319; 0.177), 0.571	0.141 (−0.107; 0.390), 0.263	−0.055 (−0.307; 0.197), 0.666
R^2^, *p*-Value	0.293, 0.003	0.221, 0.048	0.017, 0.118	0.172, 0.105

T, tocopherol; T3, tocotrienol; BW, body weight (kg), WC, waist circumference (cm), HC, hip circumference (kg), BF, body fat content (%); FM, fat mass (kg); BMI, body mass index (kg/m2), WHR, waist–hip ratio; WHtR, waist to height ratio; Σ, sum; SFA, saturated fatty acids; MUFA, mono-unsaturated fatty acids; PUFA, polyunsaturated fatty acids; TC, total cholesterol; LDL, low-density lipoprotein; HDL, high-density lipoprotein; TG, triacylglycerol; CRP, C-reactive protein; D5D, delta-5-desaturase; D6D, delta-6-desaturase; SCD16, stearoyl-CoA desaturase 16; SCD18, stearoyl-CoA desaturase; -, variables not included in the analyses; Adjusted for gender, age and smoking.

**Table 7:** Multivariate models between plasma Vitamin E isoform concentration and selected predictors.

Model	Variable	Total (n = 127)		Women (n = 77)		Men (n = 50)	
		β (95% CI), *p*-Value	R^2^, *p*-Value	β (95% CI), *p*-Value	R^2^, *p*-Value	β (95% CI), *p*-Value	R^2^, *p*-Value
α-tocopherol							
1	BF (%)	−0.201 (−0.389; −0.012), 0.037	0.221, <0.001	0.170 (−0.397; 0.283), 0.739	0.257, <0.001	−0.158 (−0.494; 0.179), 0.350	0.310, <0.001
	TC (mg/dL)	0.234 (0.073; 0.394), 0.005		0.002 (−0.201; 0.204), 0.988		0.462 (0.196; 0.727), 0.001	
	HDL (mg/dL)	0.230 (0.038; 0.421), 0.019		0.461 (0.120; 0.801), 0.009		0.081 (−0.275; 0.435), 0.652	
2	HDL (mg/dL)	0.389 (0.201; 0.578), <0.001	0.230, <0.001	0.443 (0.203; 0.684), <0.001	0.270, <0.001	0.209 (−0.096; 0.514), 0.174	0.288, 0.004
	LDL (mg/dL)	0.231 (0.068; 0.395), 0.006		0.085 (−0.118; 0.290), 0.407		0.429 (0.148; 0.800), 0.004	

β, regression coefficient; CI, confidence interval; BF, body fat content (%); TC, total cholesterol; LDL, low-density lipoprotein; HDL, high-density lipoprotein; Model 1 without adjustment. Model 2 adjustment for age and smoking as well as by gender for the total group.

## Data Availability

The original contributions presented in this study are included in the article. Further inquiries can be directed to the corresponding author.
